# The potential for eye donation from hospice and palliative care clinical settings in England: a retrospective case note review of deceased patients' records

**DOI:** 10.1007/s10561-022-10036-2

**Published:** 2022-11-02

**Authors:** Tracy Long-Sutehall, Banyana Cecilia Madi-Segwagwe, Adam Hurlow, Christina Faull, Clare Rayment, Faith Jacob, Jane Wale, Jill Short, Julie Johnston, Katerina Georgiade, Mark Brown, Naomi Seaton, Sarah Mollart, Suzie Gillon, Mike Bracher

**Affiliations:** 1 School of Health Sciences, University of Southampton, Southampton, Hampshire UK; 2 Leeds Teaching Hospitals NHS Trust, Leeds, West Yorkshire, UK; 3 LOROS Hospice, Leicester, Leicestershire, UK; 4 Marie Curie Hospice, Bradford, West Yorkshire UK; 5 Milton Keynes University Hospital NHS Foundation Trust, Milton Keynes, Buckinghamshire UK; 6 Rowans Hospice, Waterlooville, Havant UK; 7 National Health Service Blood and Transplant - Tissue and Eye Services, Speke, Liverpool, UK; 8 National Health Service Blood and Transplant - Tissue and Eye Services, Speke, Liverpool, UK; 9 West Suffolk Hospital NHS Foundation Trust, Bury, Greater Manchester, UK

**Keywords:** Potential, Eye donation, Hospice, Hospital palliative care, Retrospective note review

## Abstract

There is a need to identify additional routes of supply for ophthalmic tissue in the UK. This paper reports the findings from a national study exploring the potential for eye donation (ED) from three Hospice Care (HC) and three Hospital Palliative Care Services (HPC) in England. The objectives addressed in this paper are i.) to establish the size and describe the clinical characteristics of the potential eye donor population across six clinical sites; ii.) to identify challenges for clinicians in applying the standard ED criteria for assessing patient eligibility. Retrospective assessment of 1199 deceased patient case notes, 601 Hospice Care and 598 Hospital Palliative Care services, against current eye donation criteria. Clinicians’ assessments were then evaluated against the same criteria. by specialists based at the National Health Service Blood and Transplant Tissue Services division (NHSBT-TS). Results of the assessment and evaluation are reported as descriptive statistics (numerical data). Free-text comment boxes facilitated clarification and/or justification of review and evaluation decisions. 46% (n = 553) of 1199 deceased patients’ notes were agreed as eligible for eye donation (Hospice care settings = 56% (n = 337); Palliative care settings = 36% (n = 216). For all eligible cases (n = 553) the option of ED was recorded as being raised with family members in only 14 cases (3%). Significant potential exists for eye donation from the clinical sites in this study. This potential is not currently being realised.

## Introduction

### The need for corneal tissue—global and UK contexts

Globally, the estimated number of people who are visually impaired is reported (by WHO databases) to be 285 million, with 39 million individuals recorded as blind, and 246 million recorded as having low vision (Pascolini and Mariotti 2012). According to authors Pascolini and Mariotti (2012) over 10 million of those people reported as blind have bilateral corneal blindness, which could be restored with a corneal transplant, however these individuals do not have access to the benefits of sight saving and sight restoring transplantation surgery due to a short fall in the supply of ophthalmic tissue (cornea and sclera) that is only available via eye donation (ED).

According to the Royal National Institute of Blind (RNIB), over two million people in the UK are living with sight loss (RNIB & Specsavers 2016) caused by conditions such as Keratoconus and Fuchs’ Corneal Dystrophy, which can be treated if eye tissue is available (e.g. by corneal transplantation and reconstructive surgery). Eye tissue is also needed for research into a wide variety of eye diseases, for example, endothelial failure post cataract surgery (Pascolini and Mariotti 2012; Gain et al. 2016). The RNIB reports that approximately 5000 corneal transplants are required annually in the UK to address disease and injury resulting in sight loss, with costs to the UK economy (unpaid carer burden and reduced employment rates) reported as £4.34 billion annually (RNIB & Specsavers 2016). Critically, this organisation predicts that by 2050, the number of people with sight loss will double to nearly four million.(RNIB & Specsavers 2016) It is, therefore, imperative that the tissue needed to intervene in these conditions via corneal transplantation, reconstructive surgery, glaucoma surgery and research into the causes and treatment of eye disease is available.

## The unique and specific case of eye donation (ED)

Addressing barriers to ED require attention to the unique and specific challenges that are associated with this specific form of donation; for example, why do family members of organ donors frequently reject ED despite agreeing to other organs (and tissues)? Data from UK-based studies over the past five years indicate that personal views of potential donors and family decision makers are influential in triggering a decline when ED is proposed (Bracher et al. 2021). Prominent concerns include potential for disfigurement (Lawlor and Kerridge 2011), beliefs compatible with eyes being needed in the afterlife, and/or that eyes as ‘windows to the soul’ are an essential aspect of a person even after death (Lawlor and Kerridge 2014). Eye donation is also known to elicit specific disgust-type responses in some patients and family members, characterised as an ‘ick factor’ attended by feelings of squeamishness in respondents (O’Carroll et al. 2011), that is not observed in other forms of donation.

## Optimising the supply of ophthalmic tissue for use in sigh-saving and sight restoring surgery and medical research

The National Health Services Blood and Transplant (NHS BT) Tissue and Eye Services (TES) Bank in Speke, Liverpool, which supplies most but not all eyes for surgical purposes in the UK, aims to achieve a weekly stock of 350 eyes so that they can provide 70 eyes every working day for use in surgery or research. From April 2021 – March 2022 donation of eyes from all sources (solid organ donation, tissue donation) generated 4555 eyes from 2286 donors (National health Service Blood and Transplants 2021) equating to only 13 eyes per day and 88 eyes available per week. These levels are not supplying sufficient tissue necessary for the 5000 corneal transplants required each year for conditions such as Keratoconus, Fuchs’ Corneal Dystrophy and endothelial failure post cataract surgery (Gaum et al. 2012). The actual number of people waiting for a corneal transplant is difficult to confirm as there is no centralised waiting list for patients who need a corneal transplant (unlike in solid organ donation where there is a centralised waiting list, and the actual need may therefore be greater. A further pressure on the nationally reported donation rate of 4555 eyes is that approximately 30% of donated eyes will be discarded due to infection/viruses, with supply further compromised by a 28-day limit to storage requiring disposal of tissue thereafter.

## Current UK routes of ophthalmic tissue supply

There are currently two potential routes of supply for eye tissue in the UK:

*Route 1: Eye donation from solid organ donors—*Eye donation from solid organ donors (EPSOD) continues to prove problematic, with slow progress in increasing supply from this specific cohort of donors. For example, EPSOD generated 446 eyes between 1 April 2019 and 31 March 2020 (NHSBT 2020).

*Route 2: Eye donation from deaths outside of ICU/ED environments—*Unlike tissues such as heart valves, bone, tendons, and skin (where a cancer diagnosis is a contraindication) eye tissue can be considered for donation due to the avascular status of the cornea. Current data indicates that approx. 258,900 deaths in hospital (Office for National Statistics 2021) and 25,498 annual deaths in hospices (Office for Health Improvement and Disparities, 2020), could potentially supply eye tissue. However, from April 2021 to March 2022 NHSBT TES only received 443 referrals from 63 hospice locations with 293 of those referrals generating eyes. Therefore, on average, these 63 hospices referred seven donors to NHSBT TES. As there are 208 hospices across the UK, there is great room for improvement. Increasing supply is a key strategic aim for NHSBT TES with other professional bodies including the Royal College of Ophthalmology (Royal College of Ophthalmologists 2013), NHSBT TES OtAG (NHS-BT Organ and Tissue Advisory Group (OTAG), expressing the need for research into the barriers to eye donation and new supply routes.

## Methods

### Data collection

Eligibility for eye donation was assessed against criteria specified by NHSBT-TES (see Table 1) that constitute a list of contraindications barring the use of eye tissue in transplant operations. Our intention was for reviewers (clinical co-applicants) to assess patients against the ED criteria, and for members of the clinical support team at NHSBT TES to evaluate the assessments and confirm whether the patient was *‘eligible’*, *‘ineligible’*, or ‘*uncertain’*, for ED. Using this methodology, we could illustrate the ‘actual’ potential for ED in each research site. Each clinical principal investigator (CPI) (JS, CF, CR, SM, AH, JW) was asked to assess 200 deceased patient notes from their site, from the previous two years against these criteria. Clinical principal investigators completed a data collection proforma in Microsoft Excel. Proformas incorporated both closed responses and free-text (written) options. Free-text options aimed to identify contraindications that were particularly challenging for CPIs to assess with respect to ED eligibility (and thereby identify areas potential information and/or training needs). Clinical principal investigators then returned completed proformas to the study team (MJB) for missing data checks.

**Table 1:** Recorded contraindications by site type

Contraindication Type	Contraindication	HC (*n* = 601)		HPC (*n* = 598)		All (*n* = 1199)	
		***n***	% (cases)	*n*	% (cases)	*n*	% (cases)
Intrinsic eye disease	Acquired disorder of the eye	114	19.0	124	20.7	238	19.8
	Any ocular surgery (not including corrective laser surgery)	53	8.8	78	13.0	131	10.9
	Ocular inflammation	8	1.3	2	0.3	10	0.8
	Congenital disease/disorder of the eye	5	0.8	2	0.3	7	0.6
	Corrective laser surgery	3	0.5	2	0.3	5	0.4
	*Total Intrinsic Eye disease*	*183*	*30.5*	*208*	*34.7*	*391*	*32.6*
Neurodegenerative disorders	Diagnosed Dementia	13	2.2	86	14.3	99	8.3
	Recent onset of memory loss	30	5.0	30	5.0	60	5.0
	Diagnosed Alzheimer Disease	6	1.0	21	3.5	27	2.3
	Disease of unknown aetiology	13	2.2	12	2.0	25	2.1
	Diagnosed Parkinson Disease	1	0.2	17	2.8	18	1.5
	*Total Neurodegenerative disorders*	*63*	*10.5*	*166*	*27.7*	*229*	*19.1*
Malignancies	Lymphoma	19	3.2	22	3.7	41	3.4
	Leukaemia	7	1.2	13	2.2	20	1.7
	Myeloma	7	1.2	11	1.8	18	1.5
	Brain Malignancy with ocular involvement	12	2.0	1	0.2	13	1.1
	Malignant tumours of the anterior segment of the eye	6	1.0	1	0.2	7	0.6
	Retinoblastoma	0	0.0	0	0.0	0	0.0
	*Total Malignancies*	51	8.5	48	8.0	99	8.3
Other	Idiopathic Diseases	35	5.8	8	1.3	43	3.6
	Previous organ or tissue transplant	12	2.0	7	1.2	19	1.6
	Vasculitis	2	0.3	12	2.0	14	1.2
	Any recorded IV drug use	5	0.8	3	0.5	8	0.7
	Any recorded IVF treatment	2	0.3	0	0.0	2	0.2
	*Total Other*	*56*	*9.3*	*30*	*5.0*	*86*	*7.2*
Infections	Chronic viral hepatitis	6	1.0	3	0.5	9	0.8
	HIV infection	1	0.2	0	0.0	1	0.1
	*Total Infections*	*7*	*1.2*	*3*	*0.5*	*10*	*0.8*

## Data analysis

Proformas submitted to the study team (MJB) were then circulated for evaluation by specialist colleagues NHSBT Tissue Services (MB and JJ), using an evaluation proforma developed by the team (also completed using Microsoft Excel). Numerical data underwent descriptive statistical analysis to identify numbers and proportions of cases deemed eligible/ineligible. Data were also interrogated with respect to differences in assessment, with free-text comment boxes offering the option to comment on the decision made. All percentage figures have been rounded up or down to full numbers following the usual convention in reporting.

### Results

#### Site population and review completion characteristics

Clinical principal investigators at the six clinical sites (three Hospice Care (HC) and three Hospital Palliative Care (HPC) sites completed retrospective note review of 1199 deceased patients’ notes for patients who had died between February 2019-March 2021. Median deaths per year for all sites for this period was 429 (range = 120–1984 deaths per year). For HC settings, the median number of deaths per year was 250 (range = 120–386), and HPC median deaths per year was 513 (range = 250–1984). Notes review assessment was completed between January 2020-March 2021, with evaluation taking place between March 2020-August 2021. The mean time required for review was 21.3 min per case (SD = 45.4, range = 12.5–27).

#### Sample demographics

**Fig. 1:**
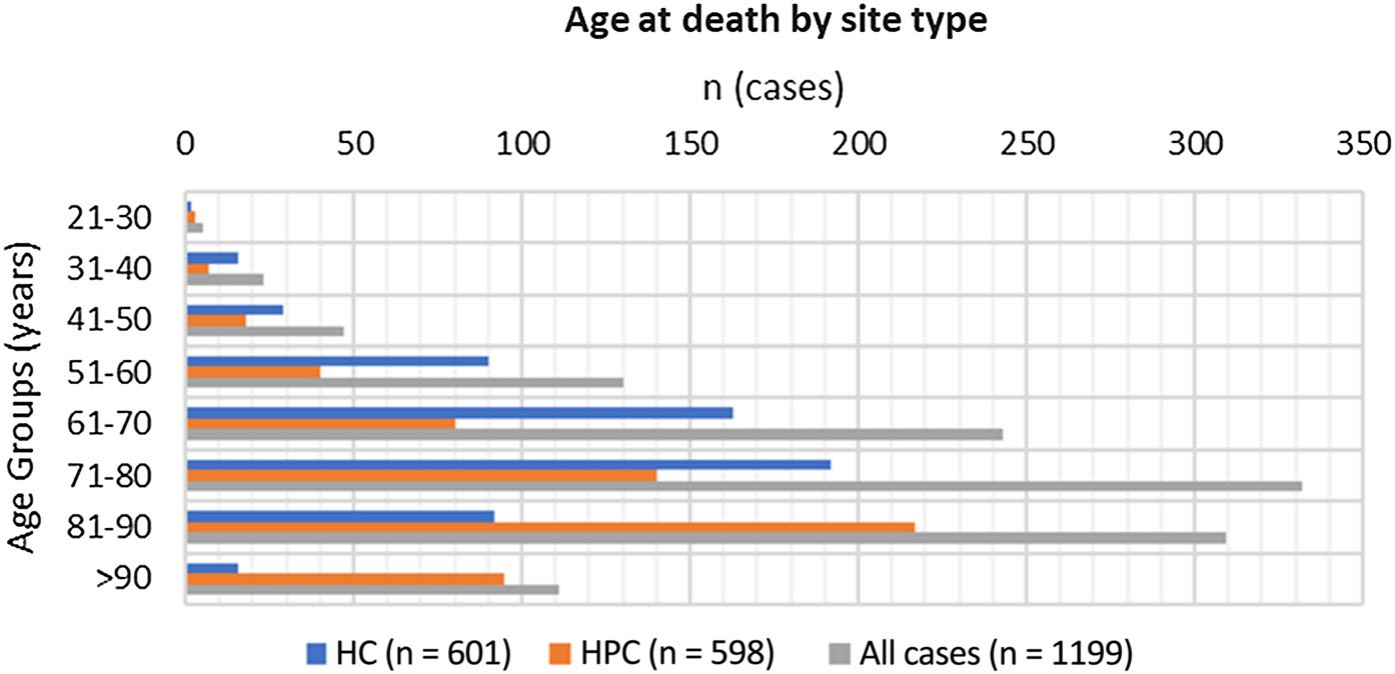
Age at death by site type

For all cases (n = 1199), mean age at death was 73.4 years (SD = 13.8; Range = 21–105) (see Fig. 1). For HC settings, mean age at death was 68.9 years (SD = 12.6; Range = 21–98), while for HPC this was 77.9 years (SD = 13.5; Range = 21–150). Female cases represented 47.9% of the total (HC = 48%; HPC = 48%). ‘White British’ was the recorded ethnicity in 82% of total cases. Ethnicity diversity was slightly higher in HC settings compared with HPC (HC = 74.0% ‘White British’ vs. HPC = 88% White British).

## Results deceased patient note review

### Agreement rate on eligibility for referral to NHSBT-TES

The total agreement rate (where assessor and evaluator made the same decision), whether *eligible*, *ineligible*, or *uncertain* re eligibility for potential eye donation was 81% (n = 972 of 1199 total case. Differences in outcome of eligibility assessments between assessors and evaluators occurred in 19% (n = 227 of 1199 total cases).

## Agreement rate on eligibility for referral to NHSBT-TES by site

Of the 601 deceased patients note reviewed in HC settings the agreement rate was 79%, (n = 475 cases) and of the 598 deceased patients’ notes reviewed in HPC settings the agreement rate was 83%, (n = 497).

## Potential for ED

Forty six percent (n = 553) of all deceased patients’ notes were agreed by assessor and evaluator as *eligible* for ED. Twenty-four percent (n = 289) of patients’ notes were agreed as *ineligible* and 11% (n = 130) were logged as *uncertain* (assessor and evaluator agreed that further information would be needed to determine eligibility).

## Potential for ED by site

Of the 601 deceased patients’ notes reviewed from HC settings, 56% (n = 337) were agreed as *eligible*, 13% (n = 77) *ineligible*, with 10% (n = 62) *uncertain*. Of the 598 deceased patients’ notes reviewed from HPC settings, 36% (n = 216) were agreed as *eligible*, 35% (n = 212) of cases *ineligible*, and 12%, (n = 68) *uncertain*.

## Record of request for ED, family approach, and referral to NHSBT from deceased patients’ notes

For all eligible cases (n = 553) the option of ED was recorded as being raised in only 14 cases (3%). Approaches to family members to discuss ED was recorded in only 13 cases (2%). Referral to NHSBT – TES for ED was recorded in 14 cases (3% of all cases) with 11 cases recorded for HC (3%) and three cases (1%) for HPC settings.

## Contraindications for ED

**Fig. 2:**
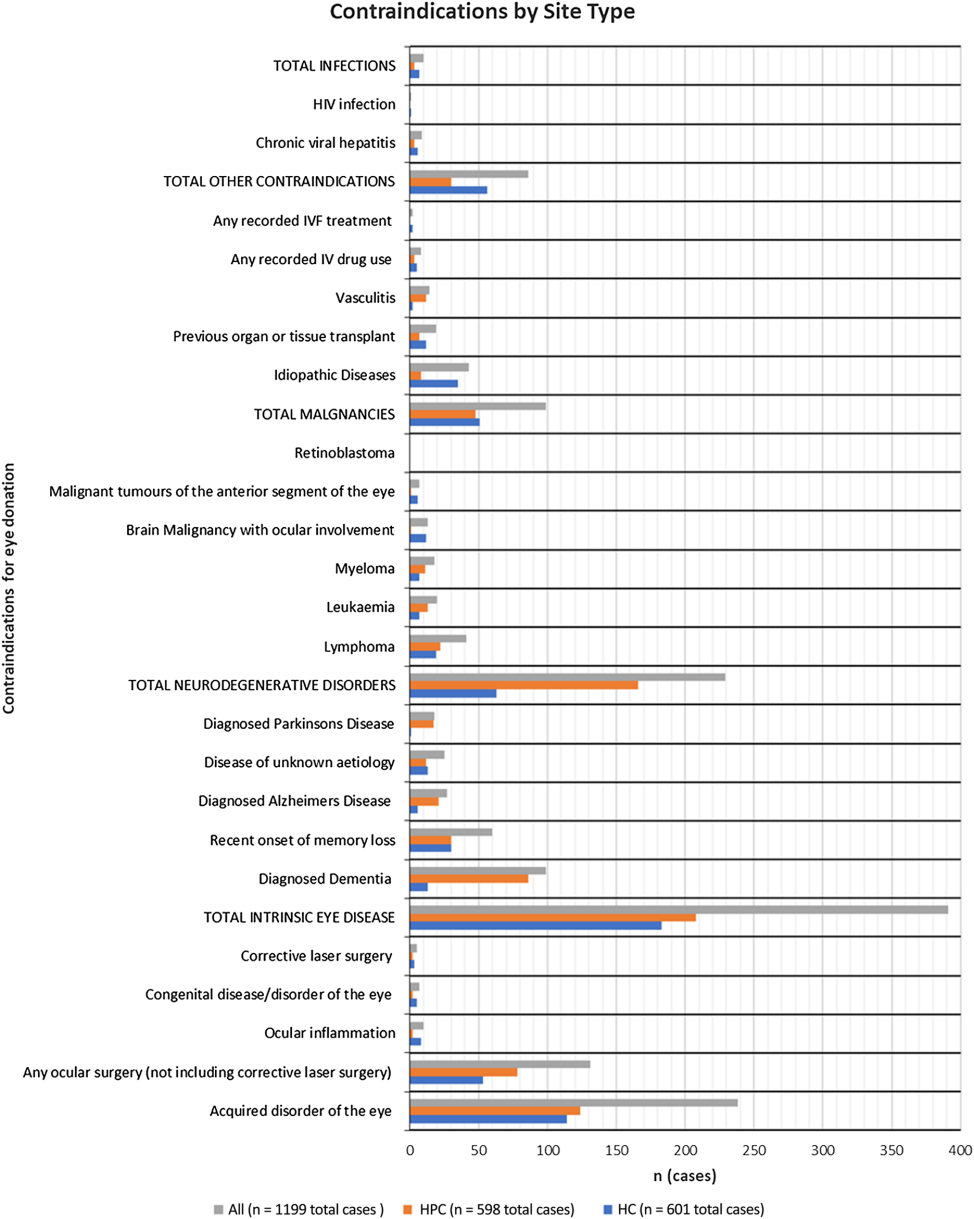
Contraindications by Site Type

For all cases, ‘Intrinsic Eye Disease’ was the most frequent contraindication reported (33%, n = 391 total cases), followed by ‘Neurodegenerative disorders’ (19%, n = 229), ‘Malignancies’ (8%, n = 99), ‘Other’ contraindications (7%, n = 86), and ‘Infections’ (8%, n = 10). Frequencies for occurrence of contraindication types across HC and HPC sites were comparable (i.e., within a < 5% range of the other), except in the case of ‘Neurodegenerative Disorders’ where the proportion of excluded cases was 17% higher in HPC settings compared with HC (11%)(see Table 1 for full reporting of contraindications and Fig. 2 for contraindications by site type).

## Differences in assessemnt for potential eye donors from HC and HPC settings from retrospective note review

This section describes numbers and types of differing assessments between clinical assessors and evaluators as a basis for identifying and clarifying the information support needs of HC and HPC staff, in assessing eligibility for ED via clinical records. Differences in outcome of eligibility assessments between assessors and evaluators occurred in 19% (n = 227/1199) of cases.

## **Differences in assessment on eligibility for*referral to*NHSBT-TES by site**

Of the 601 notes reviewed and evaluated for HC settings there was a disagreement rate of 21% (n = 125 cases), and of the 598 notes reviewed and evaluated for HPC a disagreement rate of 17% (n = 102 cases). The following sub-sections describe types of difference in assessment outcome.

## Differences in assessment outcome where the reviewer determined eligibility for referral

Of all cases, 34 (15% of total differences in assessment) involved clinical assessors determining *eligibility*, while the evaluator determined *ineligibility or uncertainty.* Of these cases, the majority (n = 28) involved miscellaneous reasons (e.g., ‘active ocular infection’, ‘Raynauds Syndrome’) assessed as not being contraindications for ED by the clinical assessor but assessed as such by the evaluator.

## Differences in assessment outcome where the reviewer determined ineligibility for referral

Forty-three percent (n = 97) of differences in assessment outcome for all cases involved clinical assessors determining *ineligibility* for ED (HC, n = 32; HPC, n = 65). Of these, 67% (n = 65) involved evaluator assessment that there were no contraindications to exclude ED referral. For example, in 31% (n = 30) of cases in this category, reviewers had excluded a patient on the basis of ocular or vision related factors (i.e., cataracts, retinopathy, ‘vision impairment due to stroke’), none of which were assessed as contraindications by the evaluator.

In 27% (n = 26) of further cases in this category the clinical assessor had assessed the patients age as exceeding the upper age cut-off for ED; however, evaluators indicated that absence of excluding ocular history or other contraindications meant the patient would be eligible for referral to NHSBT. The remaining 10% of cases in this category (n = 10) involved miscellaneous reasons for ineligibility (e.g., ‘renal transplant’, ‘confusion’) assessed by the evaluator as insufficient grounds for determining ineligibility.

## Differences in assessment outcome where the reviewer indicated uncertainty regarding eligibility for referral

Additional differences were found in 96 cases (HC = 67; HPC = 29) with differences in assessment where clinicians indicating *uncertainty* regarding eligibility for referral (e.g., ‘unsure if myelodysplasia is a contraindication’), while evaluators indicated either *eligibility* (n = 51 cases all sites; HC = 34; HPC = 17) or *ineligibility* (n = 45 cases all sites; HC = 33; HPC = 12).

In 16 cases (HC, n = 13; HPC, n = 3) evaluators determined *eligibility* while clinical assessors indicated *uncertainty* based on concerns about the eye tissue (e.g., ‘cataract surgery’, ‘ocular issues in posterior chamber of the eye’). Thirty-five further cases (HC = 21; HPC = 14) involved evaluator determination of eligibility where reviewers had indicated uncertainty for miscellaneous reasons (e.g., ‘history of asbestos exposure’, ‘Autism’).

## Cases in which no cause of death was available

In 18 cases, no cause of death was logged by the assessor (as not recorded in the patients notes).

## Discussion

The retrospective note review reported here demonstrates significant potential for eye donation across HC and HPC settings, which is currently unrealised. Across 1199 cases, 553 (46%) deceased patients notes were agreed as being eligible for ED. However, less than 3% of all cases agreed as eligible recorded an approach or referral to the relevant organisation (e.g., NHSBT – TES), therefore this ophthalmic tissue is lost from the supply chain. Our findings regarding potential are supported in the literature where authors undertaking note reviews in the UK have all identified high potential from hospice care settings (ranging from 52 and 87%, (Gillon et al., 2010; Tredgett and Ward-Davies, 2019).

Potential for donation is evidenced as higher in HC (56%) than HPC (36%) potentially influenced by patients with greater complexity and co-morbidities dying in hospital care settings. If the reported potential in HC sites (56%, n = 337) was realised this would equate to potentially 674 eyes (two eyes per donor) entering the supply chain (dependent on screening of donated tissue), and this is from just three HC sites across England.

Figures from 2020 report that there were 25,498 deaths in the 208 HC settings in the UK equating to 4.5% of all deaths in the UK (Office for Health Improvement and Disparities, 2020). However, from April 2021 to March 2022 NHSBT TES only received 443 referrals from 63 hospice locations with 293 of those referrals generating eyes. If the potential from HC sites was accessed this supply could potentially end the shortage, but not without engagement with, and investment (time, resources, finance) in Hospice Care settings identifying the key barriers that are context specific. Wider findings from the EDiPPPP have identified barriers to ED in palliative care settings and have developed an intervention *STEPS – Support Toolkit for Eye donation in Palliative care Settings* which is aimed at ensuring that the evidenced potential for eye donation from Hospice Care settings is realised and used for sigh-saving and sight-restoring transplantation and medical research.

## Data Availability

The data that support the findings of this study are not openly available due to data being from deceased patients and may be available from the corresponding author upon reasonable request.
